# MEDICAL CANNABIS USE: EXPLORING THE PERCEPTIONS AND EXPERIENCES OF OLDER ADULTS WITH CHRONIC CONDITIONS

**DOI:** 10.1093/geroni/igz038.963

**Published:** 2019-11-08

**Authors:** Lydia K Manning, Lauren M Bouchard

**Affiliations:** 1 Concordia University Chicago, River Forest, Illinois, United States; 2 Concordia University, River Forest, Illinois, United States

## Abstract

The past decade has witnessed an increased interest in the therapeutic properties of cannabis, and a growing body of research illustrates the varied uses of cannabis-based medicines for diverse symptoms, syndromes, disorder and both acute and chronic conditions, many of which are associated with advanced age (Lucas et. al, 2016). While the use of medical cannabis is on the rise in the older adult population (Kaskie et. al, 2017), more research is needed to advance the discourse on medical cannabis. With this study, we investigate older adult’s perceptions and experiences of medical cannabis use to treat and/or manage chronic conditions, specifically as a substitute for prescription drugs. Our findings suggest that older adults are open to medical cannabis as an alternative to pharmaceutical. Additionally, narratives revealed that users are hopeful that medical cannabis will provide relief with regard to the management of symptoms and relief of pain. Participants discussed their awareness and ability to manage issues related to stigma both from their primary care providers as well as family and friends. Furthermore, older adults described the frustrations with a lack of education, awareness, and support with dosing. Findings are presented as an interpretation of the participants’ perceptions of their medical cannabis use. Implications for putting medical cannabis use into everyday practice as well as policy implications are considered.

